# Understanding the selectivity *in silico* of colistin and daptomycin toward gram-negative and gram-positive bacteria, respectively, from the interaction with membrane phospholipids

**DOI:** 10.3389/fbinf.2025.1569480

**Published:** 2025-07-17

**Authors:** Yesid Aristizabal, Yamil Liscano, José Oñate-Garzón

**Affiliations:** ^1^ Grupo de Investigación en Química y Biotecnología (QUIBIO), Facultad de Ciencias Básicas, Universidad Santiago de Cali, Cali, Colombia; ^2^ Grupo de Investigación en Salud Integral, Facultad de Salud, Universidad Santiago de Cali, Cali, Colombia

**Keywords:** daptomycin, colistin, bacteria selectivity, membrane models, molecular dynamics

## Abstract

Antimicrobial resistance is a significant public health concern worldwide. Currently, infections by antibiotic-resistant Gram-negative and Gram-positive bacteria are managed using the lipopeptide antibiotics colistin and daptomycin, which target the microbial membrane. Despite the fact that both are short, cyclic, and have a common acylated group, they display remarkable antimicrobial selectivity. Colistin exhibits activity only against gram-negative bacteria, while daptomycin only against gram-positive bacteria. However, the mechanism behind this selectivity is unclear. Here, we performed molecular dynamics simulations to study the interactions between *Escherichia coli* membrane models composed of 1-Palmitoyl-2-Oleoyl-sn-Glycero-3-Phosphoethanolamine (POPE)/1-Palmitoyl-2-Oleoyl-sn-Glycero-3-Phosphoglycerol (POPG) with daptomycin and colistin, independently. Similarly, we simulated the interaction between the *Staphyloccocus aureus* model membrane composed of POPG and cardiolipin (PMCL1) with both antibiotics. We observed that colistin interacted via hydrogen bonds and electrostatic interactions with the polar head of POPE in *E. coli* membrane models, mediated by 2,4-diaminobutyric acid (DAB) residues, which facilitated the insertion of its acyl tail into the hydrophobic core of the bilayer. In *S. aureus* membrane models, weaker interactions were observed with the polar head, particularly POPG, which was insufficient for the insertion of the lipid tail into the membrane. However, daptomycin displayed strong interactions with several POPG functional groups of the *S. aureus* membrane model, which favored the insertion of the fatty acid tail into the bilayer. Contrastingly, daptomycin showed negligible interactions with the *E. coli* membrane, except for the amino group of the POPE polar head, which might repel the calcium ions conjugated with the lipopeptide. Based on these results, we identified key amino acid-phospholipid interactions that likely contribute to this antibacterial selectivity, which might contribute to designing and developing future antimicrobial peptides.

## 1 Introduction

Antimicrobial resistance is a serious public health problem due to the global emergence of multidrug resistant (MDR) bacteria because of self-medication and inappropriate use of antibiotics (Sunny et al., 2019). Infections caused by MDR Gram-negative (G-) and Gram-positive (G+) bacteria often require the use of colistin and daptomycin, respectively, which are lipopeptides considered critical “last-resort” antibiotics for managing these challenging infections (Davis and Janssen, 2020). These lipopeptides act as antimicrobial peptides, affecting the integrity and permeability of bacterial membranes (Li et al., 2021).

The cell envelopes of Gram-negative and Gram-positive bacteria possess fundamental structural differences that are key to antibiotic selectivity. Gram-negative bacteria have a complex, multi-layered envelope, featuring an outer membrane rich in lipopolysaccharide (LPS), a thin peptidoglycan layer, and a cytoplasmic (or inner) membrane. In contrast, Gram-positive bacteria lack an outer membrane but have a much thicker peptidoglycan layer and a cytoplasmic membrane that often contains teichoic acids. The cytoplasmic membranes themselves also differ in lipid composition. The *E. coli* (G-) inner membrane is primarily composed of zwitterionic phosphatidylethanolamine (PE), such as POPE, and anionic phosphatidylglycerol (PG), like POPG. The *S. aureus* (G+) membrane, however, is predominantly composed of anionic lipids, including a high concentration of POPG and cardiolipin (PMCL1), and lacks POPE (khalid, Ye y Aparicio, Simpson).

Colistin initially interacts with the LPS on the surface of G (−) bacteria and displaces divalent cations from the phosphate groups, destabilizing the outer membrane and ultimately targeting the cytoplasmic membrane (Bialvaei and Samadi Kafil, 2015). On the other hand, daptomycin depolarizes the lipid bilayer (Silverman et al., 2003) and is involved in reorganization of membrane architecture leading mislocalization of essential cell division proteins (Pogliano et al., 2012). Furthermore, perturbations of the cell membrane (CM) have been identified in daptomycin resistant strains including: extremes in CM order, resistance to CM permeabilization and reduced surface binding of daptomycin (Bæk et al., 2015).

**FIGURE 1:**
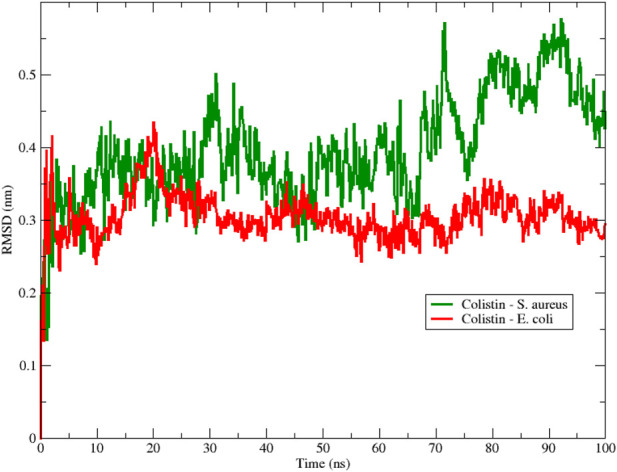
Backbone Root Mean Square Deviation (RMSD) of colistin with *E. coli* (red color) and *S. aureus* (green color) obtained from 100 ns of simulation.

Despite the fact that both lipopeptides have a completely opposite net charge, they are structurally similar and defined as cyclic peptides, comprised of a “tail” of three amino acid residues attached to a fatty acid (Huang, 2020). Particularly, colistin is a cyclic heptapeptide composed of four diaminobutyric acids (DAB), two leucines (LEU), and one threonine (THR) residues, with a linear tripeptide consisting of two DAB and one THR attached by the NH-terminal end to 6-methyloctanoic acid (Figure 1A) (El-Sayed Ahmed et al., 2020). Daptomycin is a cyclic decapeptide composed of two aspartic acids (ASP), two glycines (GLY), one serine (SER), one threonine (THR), one alanine (ALA), one ornithine (ORN), one 3-methyl-L-glutamic acid (LMG), and one kynurenine (KYN). It also contains a tripeptide consisting of an ASP, an asparagine (ASN), and a tryptophan (TRP) attached to a decanoic fatty acid by the NH-terminal end (Pokorny and Almeida, 2021).

Although both lipopeptides are structurally similar, colistin is more potent only against G (−) bacteria (Falagas et al., 2005), while daptomycin acts only against G (+) bacteria (Randall et al., 2013; Straus and Hancock, 2006). This selectivity is often attributed to the initial interaction with the outer bacterial layers; for colistin, this is its well-documented binding to LPS. However, this affinity would not be enough to destabilize and permeate the membrane, which can lead to bacterial death (Trimble et al., 2016), suggesting that subsequent interactions of colistin with the inner membrane’s phospholipids are also critical (Sabnis et al., 2021). Tristram-Nagle et al. (Dupuy et al., 2018) showed that colistin produced a softening of the membranes at an intermediate lipid/peptide molar ratio but stiffening at lower and higher peptide concentrations, whereas in G (+) and eukaryotic mimics there was only a slight softening. Additionally, X-ray and neutron reflectivity structural results reveal colistin partitions deepest to reach the hydrocarbon interior in G (−) membranes, but remains in the headgroup region in G (+) membrane, and eukaryotic mimics (Dupuy et al., 2018). Conversely, studies have shown that daptomycin has a stronger effect on membrane models mimicking G (+) bacteria employing Ca^2+^ ions (Zhang et al., 2016). Ca^2+^ at high concentrations promotes the aggregation of various daptomycin monomers to induce micelle formation, which would act as vehicles to deliver daptomycin to the surface of the bacterial cell membranes. Once in contact with the bacterial membrane, the daptomycin micelle would then dissociate, allowing monomeric daptomycin to insert into the bilayer (Taylor and Palmer, 2016). Despite multiple efforts to reveal the role of daptomycin on microbial membranes, so far the mechanism is poorly understood and controversial. Therefore, it is important to explore the specific interactions of both lipopeptides with the cytoplasmic membranes of G (−) and G (+) bacteria to determine potential structural targets that contribute to their selectivity, downstream of any initial interactions with the outer cell wall components (Malanovic, Ye y Aparicio).

While conventional methods for structural analysis, such as X-ray crystallography or NMR, provide valuable static snapshots, they often struggle to capture the dynamic and transient nature of peptide-membrane interactions. Molecular dynamics (MD) simulation offers a powerful computational alternative that provides a detailed and reliable simulation of drug-membrane interactions through time at an atomistic level. The main benefit of MD is its ability to reveal the kinetic pathways, conformational changes, and specific molecular forces that drive these interactions, offering a level of detail that complements experimental data. Its reliability has been well-established for studying the effect of antibiotics on membrane models (Liscano et al., 2019; Aragón-Muriel et al., 2021; Berglund et al., 2015; Zhao et al., 2018). Thus, in this study, we employed MD simulations to investigate the molecular underpinnings of colistin and daptomycin selectivity. We explored their interactions with simplified membrane models of Gram (−) and Gram (+) bacteria using MD simulations for 100 ns to monitor the initial approach and interaction with the membrane, and identify the participating amino acid residues.

## 2 Methods

### 2.1 3D modeling of colistin and daptomycin

The structures of colistin and daptomycin were obtained from the Protein Data Bank (PDB) with PDB ID reference 5L3G (Speranzini et al., 2016) and 1XT7 (Ho et al., 2008) respectively and then validated with the software MolProbity (http://molprobity.biochem.duke.edu/, accessed on 20 January 2023) and PROSA (https://prosa.services.came.sbg.ac.at/prosa.php, accessed on 21 January 2023). The molecules were visualized using Pymol molecular graphics system (version 2.5, Schrödinger LLC) (Yuan et al., 2017) and BIOVIA Discovery Studio Visualizer (version 2021, Dassault Systèmes, Vélizy-Villacoublay France) (Sharma et al., 2019).

### 2.2 Construction of *E. coli* and *S. aureus* membrane models

The *E. coli* and *S. aureus* membrane models were constructed using the CHARMM-GUI platform (https://www.charmm-gui.org/) and then simulated using GROMACS. Based on the typical cytoplasmic membrane composition of these bacteria, the *E. coli* model used 1-Palmitoyl-2-Oleoyl-sn-Glycero-3-Phosphoethanolamine (POPE; 80 units) and 1-Palmitoyl-2-Oleoyl-sn-Glycero-3-Phosphoglycerol (POPG; 20 units) phospholipids 19. The *S. aureus* membrane was constructed with cardiolipin (specifically, 1′,3′-bis [1-palmitoyl-2-oleoyl-sn-glycero-3-phospho]-sn-glycerol, PMCL1; 40 units) and POPG (60 units) phospholipids in both monolayers. These lipids were not derived from a single PDB entry but were built using the standard chemical definitions within the CHARMM-GUI Membrane Builder. The peptides were located at a distance of 15 Å above the membrane surface for both membrane model systems (Liscano et al., 2019). Although this model represents a simplified version of the bacterial cytoplasmic membrane, lacking components such as proteins, carbohydrates, lipoteichoic acid (for Gram (+) bacteria), and, critically, LPS for Gram (−) bacteria, the simulations provide valuable insights into the specific peptide-phospholipid interactions that occur after the peptide has traversed the outer layers. This focus allows us to isolate and analyze the contribution of the cytoplasmic membrane lipids to antibiotic selectivity.

### 2.3 Molecular dynamics simulation

The MD simulations were performed with GROMACS version 2022.2 (Abraham et al., 2015). The water box system was recreated using a TIP3P water model, as implemented in GROMACS and recommended for use with the CHARMM36m force field, for a total of 19,557 water molecules. The medium was solvated with 0.15M calcium chloride (CaCl_2_) (for daptomycin and colistin) using the Monte Carlo method. A single peptide molecule (colistin or daptomycin) was added for each bacterial system. The CHARMM36m force field was used at a temperature of 310 K, and the ensemble was created using the NPT method. The simulation of the peptide-membrane system in solution was divided into 3 phases: 1) minimization, 2) equilibrium and 3) production. For the minimization of the system, the Verlet cutoff scheme was adopted with the Steepest descent algorithm converging to a minimum energy value of 1000 kJ mol^−1^ nm^−1^ for 5000 steps at 1 fs/step. The Particle Mesh Ewald (PME) method was used to efficiently calculate the long-range electrostatic potentials. The equilibrium process was subdivided into 6 sub-stages where the first three stages were performed at a rate of 1 fs/step for 125 ps and the last three stages at a rate of 2 fs/step for 500 ps, using the jump integration algorithm to integrate Newton’s equations of motion, using Berensen’s method (Berendsen et al., 1984). MD simulations were performed for 100 ns with Particle Mesh Ewald and the SHAKE algorithm with 2 fs intervals (Harvey and De Fabritiis, 2009). In subsequent analyses, we determined the root mean square deviation (RMSD) and the most frequent intermolecular interactions between phospholipids along with the lipopeptides of colistin and daptomycin. The RMSD calculation was obtained by command with the Gromacs package, between Backbone-Backbone. The interactions were obtained in Discovery Studio for each 2ns sampling, whose data were transformed in the R Studio program to obtain Alluvial plots using the ggplot2 and ggalluvial libraries. For the mapping of the total interactions in the peptide structures, the total frequency of interactions was counted in a heatmap type plot (Yuan et al., 2017; Sharma et al., 2019; Ihaka and Gentleman, 1996). The RDF values were obtained for each simulation model using the same GROMACS package. For calculating the radial distribution, the membrane phospholipids and the lipopeptides of Colistin and Daptomycin were set as the reference center, while the radial distancing was conducted around the ions in solution (Abraham et al., 2015). The *covar* module from the GROMACS package was used to obtain the covariance matrix of the atomic positions of the backbone from the 100 ns trajectory of the 4 simulated systems. Additionally, the *anaeig* module from the GROMACS package was used to obtain the eigenvectors and eigenvalues of the previously calculated covariance matrix to perform the principal component analysis (PCA). Finally, the *sham* module from the GROMACS package was used to generate two-dimensional free energy landscape (FEL) (Abraham et al., 2015).

## 3 Results and discussion

### 3.1 Colistin selectivity toward *Escherichia coli*

It is well established experimentally that colistin is potent against Gram (−) bacteria like *E. coli* (MIC ≈0.06 mg/L) but largely inactive against Gram (+) bacteria such as *S. aureus* (MIC >64 mg/L) (Klinger-Strobel et al., 2017). To investigate the molecular basis of this selectivity at the cytoplasmic membrane level, we first analyzed the conformational stability of colistin upon interacting with our model membranes.

To assess the structural stability of the peptide during the simulation, Figure 1 shows the RMSD obtained for the interactions between colistin and the *E. coli* and *S. aureus* membrane models using 100 ns MD simulations. From the beginning of the simulation, we observed that the RMSD fluctuated for both membrane-peptide systems, suggesting that they are highly dynamic systems, reflecting only large displacements of a small structural subset within an overall rigid structure (Aragón-Muriel et al., 2021). Considering the aforementioned, a smaller amplitude in the oscillation range is interpreted as greater structural stability associated with the conformation at each moment in time, due to an increase in conformational rearrangements that better adapt to the surrounding environment; that is, the interactions occurring between the studied molecular structure and other components, both in solution and with macromolecules, exert their effect, limiting the number of conformational changes that would allow the system to reach thermodynamic equilibrium with its environment. A notable range of oscillation and a lower average RMSD value are evident for the *E. coli* model, in contrast to the *S. aureus* model, which exhibited higher values in both proposed scenarios regarding conformational stability. This result provides initial insight into the stability of colistin when interacting with membrane models, suggesting that colistin achieves a more stable conformation when interacting with the *E. coli* model membrane.

**FIGURE 2:**
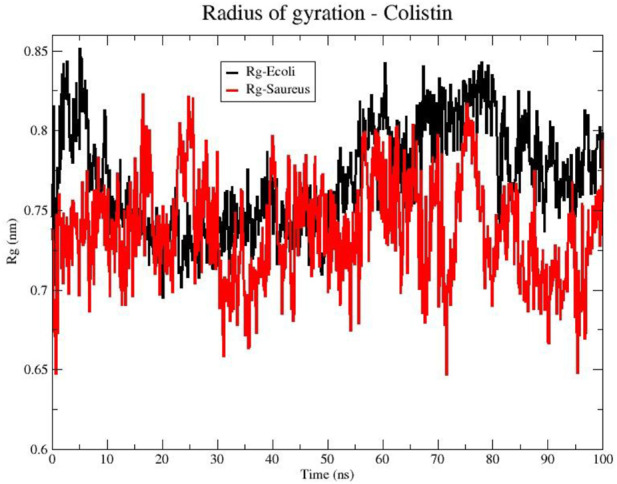
Radius of gyration (Rg) of the Colistin structure in bacterial models of *E. coli* (black line) and *S. aureus* (red line).

To further evaluate the compactness of colistin’s structure upon membrane interaction, the radius of gyration (Rg) is a measure of the size distribution of a molecule in the space. In Figure 2, the Rg of colistin is shown for two membrane models *(E. coli* and *S. aureus*). The line representing colistin in the *E. coli* (black color) fluctuates over time, suggesting changes in its conformation. However, from 20 ns the fluctuations are smaller compared to that exhibited with the *S. aureus* model. This could indicate a more compact or closer interaction of colistin with the *E. coli* membrane, possibly increasing its effectiveness in pore formation in that membrane since the peptide radius of gyration decreases from bulk water to bilayer core (Hu et al., 2015). On the other hand, the line representing colistin in the *S. aureus* membrane (Figure 2, red color) also shows variability in the Rg over time. However, there is a trend towards lower Rg values exhibiting greater variability in the interaction with the phospholipids of the *S. aureus* membrane compared to *E. coli*. Collectively, these Rg results suggest that colistin adopts a more compact and stable structure on the *E. coli* membrane, whereas its interaction with the *S. aureus* membrane is more variable and less stabilized.

**FIGURE 3:**
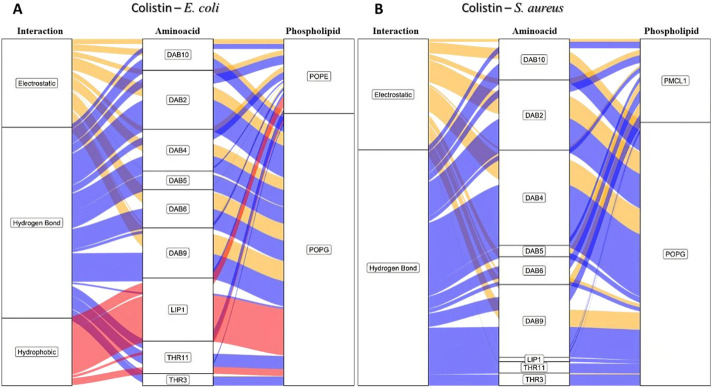
Alluvial diagram of the interactions between the colistin amino acid residues and the membrane system of **(A)** *E. coli* and **(B)** *S. aureus* in 100 ns simulation. The electrostatic type interactions were associated with *yellow* fluxes, the hydrogen bond type with *blue* fluxes and the hydrophobic type with *red* fluxes.

In order to identify the residues on colistin that are involved in the RMSD fluctuations and the types of interactions involved, we developed an alluvial diagram (Figure 3) representing the count by interaction type for each amino acid residue in colistin on each phospholipid, for the *E. coli* and *S. aureus* membrane models. Hydrogen bonding interactions were predominantly observed between the organic functional groups, such as amino, hydroxyl, carbonyl, and carboxyl residues, in the polar heads of the phospholipids in both membrane models and the colistin residues. The amino acid residues in both bacterial models that most frequently participated in these interactions were 2,4-diaminobutyric acid (DAB) and threonine (THR). The side chain of DAB contains a protonated amino group with a residual positive charge which forms hydrogen bonds and electrostatic interactions. Meanwhile, THR has a hydroxyl group capable of forming hydrogen bonds. Hydrogen bonds are primarily formed with POPE (Figure 3A), possibly because POPE is abundant in these membrane models. A study comparing the selectivity of peptides toward POPE to that toward anionic lipids concluded that the affinity toward POPE might be because the binding pocket of the primary amine on POPE’s polar head is smaller than the surface of the peptide, which cannot accommodate larger polar head groups (Hosoda et al., 1996).

The difference between hydrogen bonding interactions in the two bacterial membrane models is mostly due to the type of phospholipid involved. With POPE and POPG being the main phospholipids for *E. coli*, the selectivity was mainly due to the substitution of a hydroxyl group for an ammonium group in the polar head of the phospholipid, resulting in a greater number of hydrogen bond formation in the *E. coli* bacterial model. Contrastingly, in *S. aureus*, hydrogen bonds were observed more frequently with POPG compared to PMCL1 (Figure 3B) despite the insignificant difference in the number of molecules between the two phospholipids. These results might be attributed to their structural properties. Cardiolipin comprises two POPG molecules and a single hydroxyl in the polar head, while POPG has two hydroxyls with greater hydrogen bonding capability (Rocha‐Roa et al., 2021; Hernández-Villa et al., 2018).

As the system is at a neutral pH, it favors the protonation of the DAB amino group, whose positive charge strongly dominates the nitrogen atom. Hence, this functional group is frequently involved in electrostatic interactions (the second most frequent interaction for both bacterial membrane models), primarily with the anionic phosphate groups of the phospholipids (Travkova et al., 2017; Balleza et al., 2019). Hydrophobic interactions might also contribute to the selectivity as they occur more frequently in the *E. coli* bacterial model than in the *S. aureus* model (Figure 3). THR is indispensable for these hydrophobic interactions. The lateral hydroxyl group might facilitate the orientation of the phospholipid acyl chains towards an adjacent amino acid residue to the hydroxyl group containing a methyl group interacting with the hydrocarbon chain of the phospholipids on the surface of the membrane model (Kralt, 2020; Cirioni et al., 2013). Interestingly, hydrophobic interactions were only observed between the colistin lipid chain and the *E. coli* model (Figure 3A), which might determine the antimicrobial selectivity against Gram-negative bacteria. This analysis reveals that specific interactions, particularly hydrophobic ones involving the lipid tail, are unique to the colistin-*E. coli* model system, pointing to a potential mechanism for selectivity.

**FIGURE 4:**
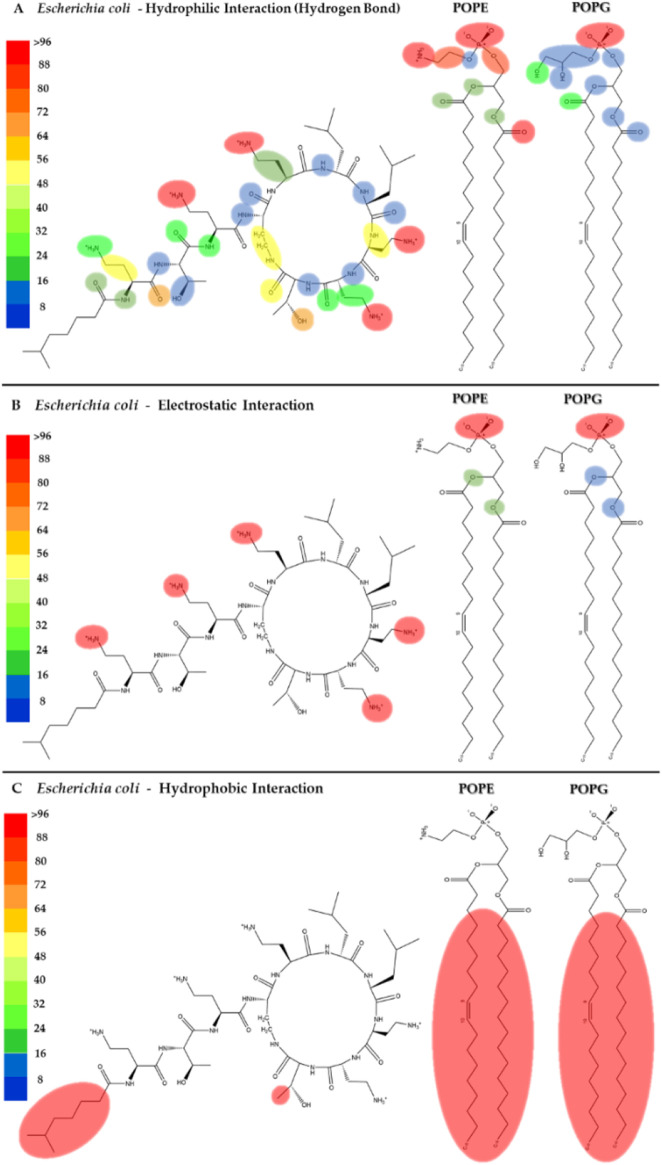
Heatmap of the interactions of colistin with the POPE and POPG phospholipids in the *E. coli* membrane model. The interaction frequency scale ranges from blue (lowest number of interactions) to red (highest number of interactions) **(A)**: Hydrophilic interactions–*E. coli*; **(B)** Electrostatic interactions–*E. coli*; **(C)** Hydrophobic interactions–*E. coli*.

In order to comprehend this phenomenon, we generated a heatmap based on the three types of interactions in the *E. coli* model (Figures 4A–C). Here, the amino groups of DAB and of the polar head of POPE would act as donor groups, while the uncharged oxygen of the phosphate would serve as the acceptor group in the hydrogen bond (Figure 4A). Furthermore, the heatmap shows that the amino groups of the lateral chain of the DAB were properly protonated at pH = 7 and exhibited more electrostatic interactions due to a ionic charge (Figure 4B). They also contribute to the net charge of +5 in the colistin molecule and provide the most hydrogen bonding interactions. The hydroxyl (-OH), carbonyl (-C=O), and amide (-(C=O)-NH-) groups present in the molecule contributed to a lesser extent but with equal relevance in the total stabilization of the peptide within the bilayer. Moreover, the single threonine (THR) residue located at the cyclic head of the polypeptide and the 6-methylheptanoic acid (LIP) contributed to the hydrophobic interactions (Figure 4C). The insertion of a peptide into the lipid bilayer disrupts the acyl chains (Supplementary Figure S1), which decreases the bilayer’s capacity to act as a barrier. This process is thought to allow the release of cytoplasmic content, eventually causing bacterial death (Wang et al., 2020).

**FIGURE 5:**
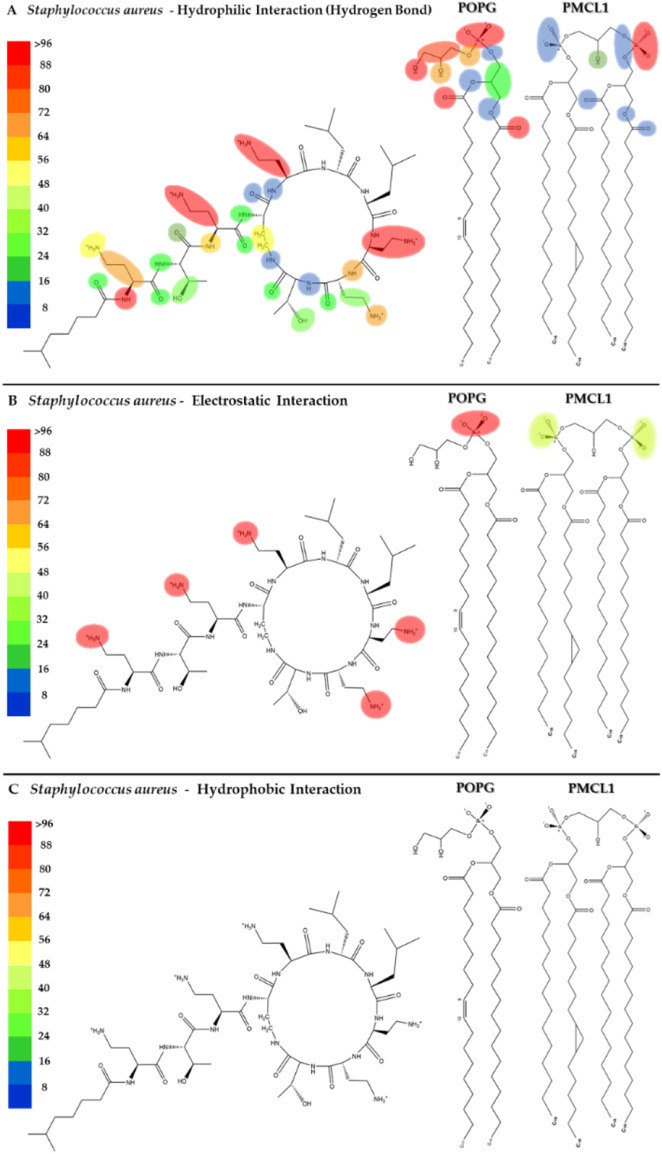
Heatmap of interactions of colistin with POPG and PMCL1 in the *S. aureus* membrane model. The interaction frequency scale ranges from blue (lowest number of interactions) to red (highest number of interactions) **(A)**: Hydrophilic interactions–*S. aureus*; **(B)** Electrostatic interactions–*S. aureus*; **(C)** Hydrophobic interactions–*S. aureus*.

Figures 5A–C shows the heatmap for the intermolecular hydrogen bond, electrostatic and hydrophobic interactions of colistin with the POPG and PMCL1 phospholipids in the *S. aureus* membrane model. The functional groups present in the colistin amino acid residues, including the amino, carbonyl, and hydroxyl groups, contributed equally to the hydrogen bond interactions with the *S. aureus* membrane model but to a lesser extent compared to the *E. coli* membrane model (Figure 5A). On the other hand, although electrostatic interactions were mainly observed between the amino (-NH_2_) groups of the alkyl chain of DAB in colistin and the *S. aureus* membrane model (Figure 5B), this was lower compared to the *E. coli* model. The magnitude of all these interactions was not sufficient for the insertion of the colistin lipid tail into the hydrophobic core of the bilayer (Figure 5C). This finding is critical, as it suggests that the weaker interactions with the *S. aureus* membrane lipids prevent the crucial step of hydrophobic tail insertion. This might be because the enthalpic contributions of each of these interactions were inadequate to compensate the entropy of the colistin acyl tail within the polar region of the phospholipid, preventing it from assuming an appropriate orientation toward the hydrophobic core of the bilayer. Additionally, some structural properties of the phospholipids might also influence the insertion of the lipid tail. PMCL1 has a small polar head group in proportion to the volume occupied by its four hydrocarbon chains, meaning fewer polar groups per unit area unlike two POPG molecules. The two phosphates in the head group recruit a proton and are locked into a bicyclic matrix bonded by the hydroxyl residue of glycerol, connecting the two-halves of the PMCL1 molecule. This small polar head group causes tighter packing of the hydrophobic acyl chains between the PMCL1 molecules due to Van der Waals interactions compared to those found in lipids with larger polar head groups (Matsumoto et al., 2006), opposing the insertion of the colistin lipid tail.

The POPG molecule, constituting 58% of the bacterial models, showed interactions with the carbonyl, hydroxyl, and phosphodiester groups. Due to the presence of an atom with free electron pairs, it acts as a donor during the formation of hydrogen bonds. However, the polar head interactions are much lower than those observed in the *E. coli* membrane model (Figures 4, 5). POPG phosphate groups had a balance between interactions with water and with the glycerol moieties of adjacent lipids. In fact, phosphates have the closest interactions with glycerol groups from very strong H-bonds (Elmore, 2006), which promotes the exclusion of PMCL1 molecules generating PMCL1-aggregates in the membranes (Matsumoto et al., 2006). A less dynamic and more compact interface structure could decrease the membrane permeability (Zhao et al., 2008), thus affecting the antimicrobial activity of colistin against *S. aureus* (Supplementary Figure S2). Therefore, the combination of weaker peptide-lipid interactions and the intrinsic physical properties of the *S. aureus* model membrane appears to create a barrier to colistin’s action.

**FIGURE 6:**
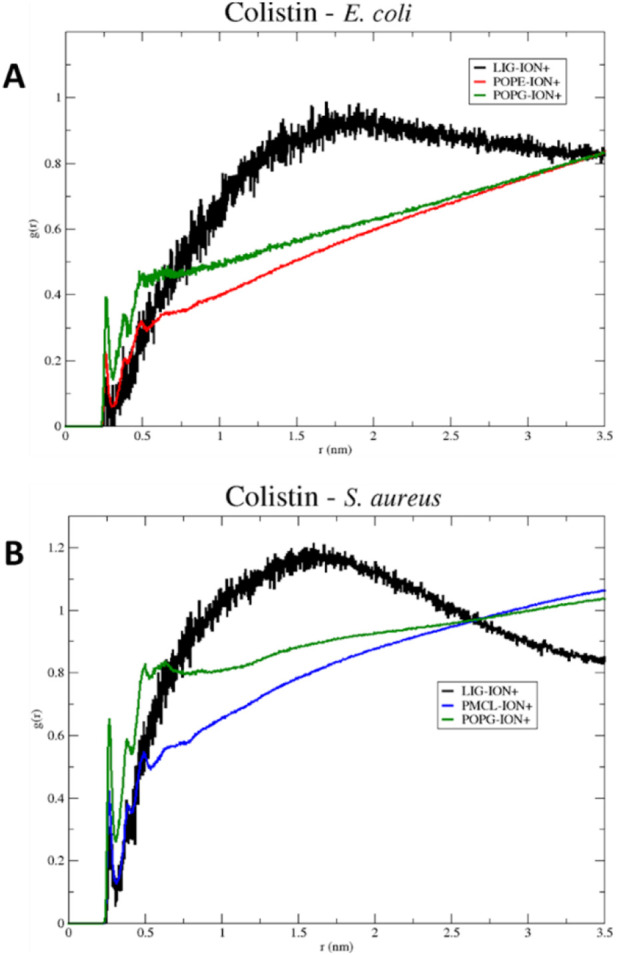
RDF (Radial Distribution Function) of colistin in models **(A)** *E. coli* and **(B)** *S. aureus*, for a 100 ns simulation.

To explore the influence of the ionic environment on these interactions, in Figure 6, the radial distribution diagrams for the membrane models of *E*. *coli* (Figure 6A) and *S. aureus* (Figure 6B) are observed. The distribution of positive ions (mobile molecules) over colistin and the phospholipids present in each membrane as reference molecules (static molecules) is visualized radially. Initially, the first peak observed in each diagram reveals the affinity of the ions towards each of the static structures. The membrane phospholipids, characterized by possessing a negative charge density on their surface, create a greater gradient of positive ions over the same surface, in contrast to the structure of colistin, which is characterized by being cationic in nature. As the distance increases, the local relative density of the ions approaches the average density of the system, resulting in a generalized increase in the radial distribution of ions in solution. However, the radial distribution function for colistin reaches a maximum before decreasing, due to the displacements occurring in the water box over time. The point where the radial distribution functions of the membrane phospholipids intersect with that of colistin corresponds to the equalization of ion densities between the membrane structures and colistin.

For the *S. aureus* model, the crossing point occurs more rapidly than in the *E. coli* model. Therefore, this may limit its permeabilizing action on the *S. aureus* membrane model, as it encounters an electrostatic potential barrier that may repel the cationic structure of colistin. Cationic ions in solution can prevent colistin from stabilizing on the membrane surface, as they have a greater capacity to solvate on more negative surfaces, such as that of the *S. aureus* model. This result clarifies an additional mechanism by which colistin’s selectivity may be influenced: its interaction with the membrane surface must be strong enough to overcome the competing electrostatic cohesion between solvated ions and the membrane itself.

**FIGURE 7:**
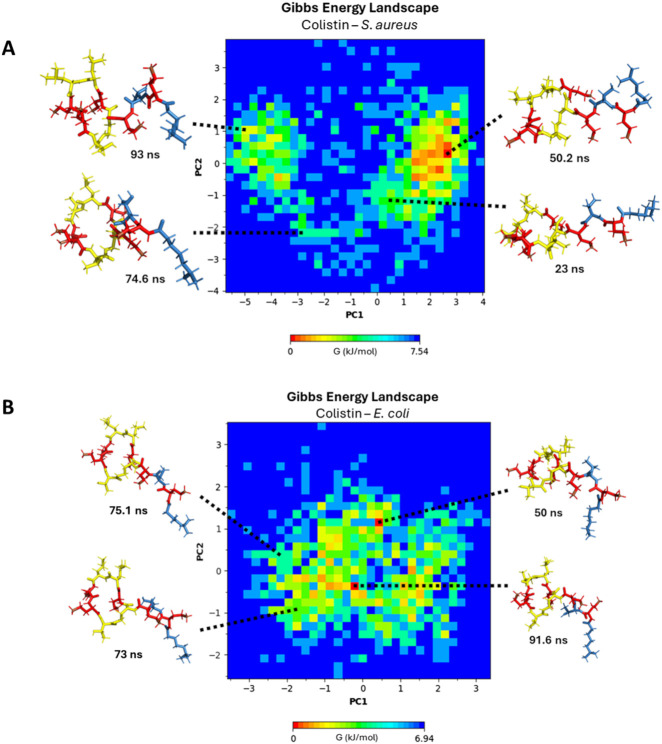
Gibbs free energy diagram using principal component analysis (PCA) **(A)** *E. coli* membrane model system. **(B)** *S. aureus* membrane model system. DAB residues: red color, linear chain: blue color, cyclic chain: yellow color.

For a more suitable analysis of the approach of colistin and daptomycin lipopeptides to bacterial membrane models, the most favorable conformation can be revealed in terms of Gibbs free energy. For this purpose, the PCA method allows for the dimensionality reduction of large datasets calculated in simulation trajectories, reducing the noise level of data that contributes the least to their interpretation (Kurita, 2020). In the end, a pattern of structural conformational changes in these lipopeptides is obtained, enabling a two-dimensional plot (the first two principal components) of the simulation system’s behavior, transforming thermodynamic data into free energy landscape (FEL) (Greenacre et al., 2022; Maisuradze et al., 2009). In Figure 7A, a Gibbs free energy profile diagram is presented using principal component analysis (PCA) to reveal the structural conformational changes of colistin positioned on the *E. coli* membrane model. For the conformations shown at 50, 73, 75.1, and 91.6 ns, the cyclic fragment of colistin is oriented perpendicularly to the membrane. Moving from left to right along the X-axis of the energy diagram, the orientation of the linear chain changes from an extended form aligned parallel to the membrane surface to one where the folding of the linear chain allows it to position itself perpendicularly to the surface, as observed in the sampled conformations. In contrast, the variation along the Y-axis from bottom to top represents the cyclic structure, transitioning from a fully extended ring to a ring that folds inward toward its center. For the most stable conformation, as seen at 50 ns and 91.6 ns, the insertion of the lipid tail is stabilized by hydrophobic interactions within the membrane, accompanied by electrostatic interactions and hydrogen bonding from the DAB residues. The insertion of the lipid tail facilitates the cyclic fragment’s ability to fold inward more easily than if it were not inside, as observed in the samples at 73 ns and 75.1 ns. In the latter case, the inward folding of the ring is not achieved, as the hydrophobic interactions of the lipid tail only accommodate part of the lipid chain.

In Figure 7B, a Gibbs free energy profile diagram is shown using principal component analysis to determine the structural conformational changes of colistin after interacting with the *S. aureus* membrane model. At 23, 50.2, 74.6, and 93 ns, the cyclic fragment of colistin is oriented perpendicularly to the membrane. Moving towards the extreme values from zero along the X-axis of the energy diagram, the orientation of the linear chain changes from an extended conformation to a retracted conformation at the extreme values of PC1. On the other hand, the variation that occurs on the Y-axis, on the cyclic structure towards the extreme values (more positive or more negative) of the PC2 component, the cyclic structure extends in a planar manner, while towards values close to zero the cyclic structure retracts towards the center. At the extremes of component PC2, the cyclic structure extends in a planar manner, while values closer to zero indicate that the cyclic structure retracts toward the center. For the most stable conformation at 50.2 ns, the retracted form of the lipid tail oriented away from the membrane surface demonstrates an improvement in stability during its approach. The approach of colistin towards a membrane surface with high ion solvation repels the lipid tail’s proximity to the membrane to a greater extent, as evidenced in the RDF diagrams (Figure 6B). The free energy landscape clearly illustrates that colistin fails to achieve a stable, inserted conformation in the *S. aureus* model, in stark contrast to its behavior with the *E. coli* model.

These findings are integrated with experimental results reported in other studies. For example, Klinger et al. (Klinger-Strobel et al., 2017) reported a colistin MIC for *E. coli* of 0.0625 mg/L, in contrast to *S. aureus*, which exhibited an MIC of >64 mg/L, consistent with observations in the research by Dupuyi etl al. (Dupuy et al., 2018). That study revealed that colistin disrupts only the membrane of Gram-negative bacteria by inserting into the hydrophobic core, whereas in Gram-positive bacteria, it remains in the polar region.

The results for colistin indicate that its selectivity for the Gram-negative *E. coli* model membrane is driven by strong electrostatic and hydrogen bonding interactions, primarily between its cationic DAB residues and the POPE phospholipids. These interactions stabilize the peptide on the membrane surface, facilitating the crucial insertion of its hydrophobic tail into the lipid core. In contrast, its interaction with the *S. aureus* model membrane is significantly weaker and more transient, failing to promote tail insertion. This difference in interaction at the phospholipid level provides a computational rationale for its observed biological selectivity.

### 3.2 Selectivity of daptomycin toward *S. aureus*

Although it has been widely documented that daptomycin forms micelles consisting of 14–16 monomers for each equivalent of Ca^2+^ present (Ho et al., 2008; Ball et al., 2004), It has been suggested that daptomycin acts on membranes in a monomeric state when the concentration is between 0.02 and 0.75 µM. (Zhang et al., 2017). Furthermore, in a solution of the physiological Ca^2+^ concentration (∼1 mM) daptomycin is in monomeric state below 0.5 mM. Therefore, at the therapeutic concentration (<50 μM) daptomycin is monomeric in solution (Huang, 2020). Thus, to understand the interaction of daptomycin with phospholipids it is relevant to consider the molecular state that daptomycin has at therapeutic concentrations.

**FIGURE 8:**
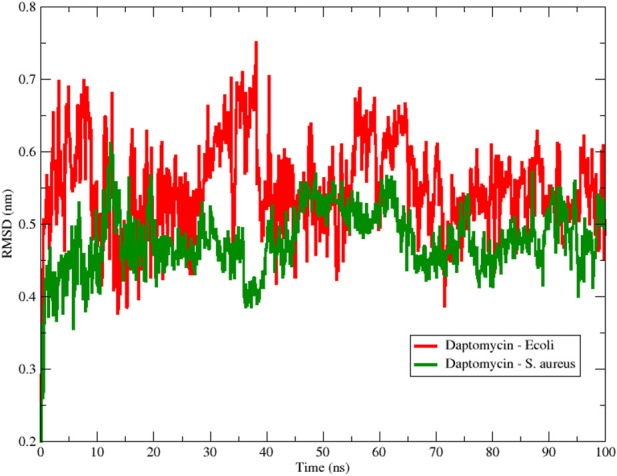
Backbone Root Mean Square Deviation (RMSD) of daptomycin with *S. aureus* (red color) and *E. coli* (green color) membrane models using 100 ns simulation.

To begin the analysis, we evaluated the conformational stability of daptomycin on both membrane models. In Figure 8, the root mean square deviation (RMSD) of the daptomycin structure is observed during its approach and interaction with the membrane models of *E. coli* and *S. aureus*, for a simulation time of 100 ns. A notable oscillation range and a lower average RMSD value for the *S. aureus* model are highlighted, in contrast to the *E. coli model*, which was higher in both scenarios. A smaller amplitude in the oscillation range is interpreted as greater conformational structural stability with the interacting medium, due to an increase in conformational rearrangements that better adapt to the surrounding environment. This means that the interactions occurring between the studied molecular structure and other components, both in solution and macromolecules, exert their effect on it, limiting the number of conformational changes that allow for achieving thermodynamic equilibrium with the surrounding environment (Zhao et al., 2008; Kurita, 2020). Thus, these results provide an initial insight into the stability of daptomycin during its approach and interaction with the studied membrane models, suggesting that the combined chemical nature of the *S. aureus* phospholipids is capable of inducing a more stable conformation in daptomycin.

**FIGURE 9:**
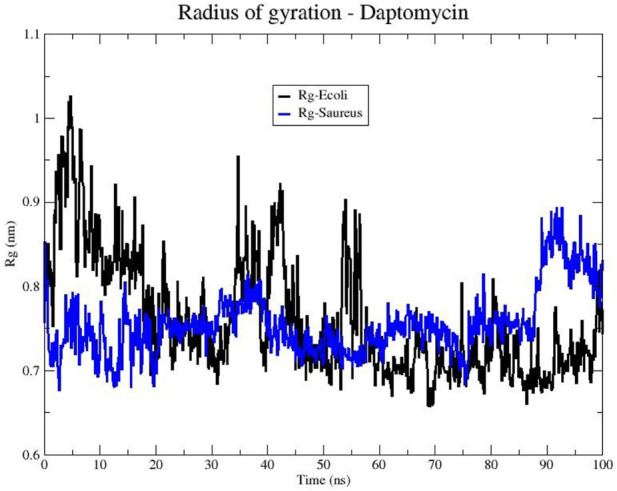
Radius of gyration (Rg) of the Daptomycin structure in bacterial models of *E. coli* (black line) and *S. aureus* (blue line).

Next, we examined the peptide’s compactness to complement the stability analysis. Figure 9 shows the changes in the Rg of daptomycin when it interacts with membranes of *E. coli* and *S. aureus* over time. The line representing daptomycin in the *S. aureus* membrane (blue color) shows variability. However, the Rg appears to remain relatively constant over time. This suggests that daptomycin may adopt a moderately stable conformation maintaining a constant dynamic pattern in the *S. aureus* membrane during the simulated time. On the other hand, the line representing daptomycin in the *E. coli* membrane (black color) shows greater variability in the Rg, in contrast to that obtained in *S. aureus* model. These results suggest that daptomycin could adopt several transient conformations in the *E. coli* membrane, reflecting a more dynamic interaction with this membrane.

These differences in daptomycin’s behavior in the two membranes could be indicative of variations in its interaction with different types of bacteria. For instance, these results suggest daptomycin has a higher affinity and a more effective, stable interaction with the *S. aureus* membrane, which could correlate with its increased antibacterial activity against this bacterium.

**FIGURE 10:**
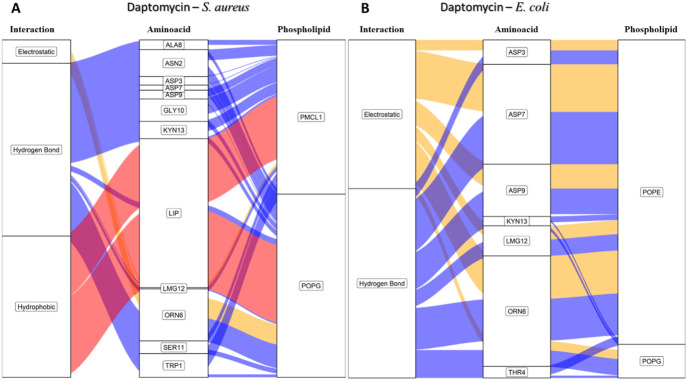
Alluvial diagram of the interactions between the amino acids of daptomycin and the membrane system of **(A)** *S. aureus* and **(B)** *E. coli* using 100 ns simulations. The electrostatic type interactions were associated with *yellow* fluxes, the hydrogen bond type with *blue* fluxes and the hydrophobic type with *red* fluxes.

To comprehend these findings, we conducted approximations of the interactions that govern peptide stability on the surfaces of bacterial membrane models employing an alluvial diagram showing the proportion based on the type of interaction between each amino acid residue of daptomycin and each phospholipid from the *S. aureus* and *E. coli* membrane models (Figure 10). Hydrogen bond interactions were the most frequent for both bacterial membrane models, followed by electrostatic interactions. Crucially, hydrophobic interactions associated with inserting the lipid tail of daptomycin into the interior of the membrane were only present in the *S. aureus* membrane model, similar to that seen for colistin with the *E. coli* membranes.

For the *S. aureus* membrane model, the proportion of interactions with POPG and PMCL1 were similar, slightly higher for PMCL1, and their contribution to the stabilization of daptomycin was equally crucial for the lipid tail insertion. Figure 10A shows that aspartic acids (ASP3, ASP7, ASP9) and 3-methyl-L-glutamic acid (LMG12) did not contribute to the electrostatic interactions with phospholipids, suggesting that repulsions might occur between the deprotonated carboxylate groups and the phosphates of the phospholipids. However, these anionic groups are indispensable for interactions with Ca^2+^, which mediates the binding between daptomycin and the membrane (Jung et al., 2004). Additionally, these residues moderately participated in the hydrogen bond interactions with the hydroxyl groups on the polar head of POPG and PMCL1. Nonetheless, the main contributors to this type of interaction were the amino acid residues, including tryptophan (TRP1), asparagine (ASN2), ornithine (ORN6), serine (SER11), and kynurenine (KYN13). These have free amino groups that can form hydrogen and electrostatic bonds, although the latter are in smaller proportion (Figure 10A). Hydrogen bond interactions were also observed with alanine (ALA8) and glycine (GLY10). Although they do not possess any electronegative groups that can promote these interactions, the anomeric carbon flanked by two electronegative groups allows it to instantaneously form prolonged dipoles that favor this interaction, which the simulation system is capable of registering in its hydrogen bond formation measurements. The only organic groups capable of forming hydrogen bonds are those involved in peptide bonds and are perhaps responsible for generating this type of bond when approaching the surface of the lipid membrane. A study reported that hydrogen bonds could emerge between the backbone carbonyls of some residues and phospholipid polar heads (Vestergaard et al., 2019). Interestingly, hydrophobic interactions were observed between the acylated tail of daptomycin and both phospholipids, which might cause bacterial death, as it has been reported that daptomycin kills bacteria by permeabilizing and depolarizing the membrane (Zhang et al., 2016). The presence of these hydrophobic interactions exclusively in the *S. aureus* model is a key finding that computationally supports its selective mechanism.

In the *E. coli* membrane model (Figure 10B), the only interactions present in the simulation with daptomycin were hydrogen bonds and electrostatic interactions. These interactions mainly occurred with the POPE molecules, suggesting that the anionic side chains of aspartic acid and methionine might interact with the positive amino group of POPE. Conversely, the *S. aureus* membrane model (Figure 10A) showed weak interactions with the membrane surface, except presumably with cations in solution. In *E. coli* membranes, hydrophobic interactions were not found, as the interactions of the daptomycin structure failed to stabilize its approach to the polar head of the phospholipids. This hindered the entry and coupling of the daptomycin lipid into the interior of the membrane model. This might contribute to its selectivity with this bacterial model, as daptomycin is more inclined to interact with membrane models with more cationic phospholipids. The resistance of *S. aureus* to daptomycin is reportedly associated with an increase in the conversion of POPG to lysyl-POPG, a cationic phospholipid (Friedman et al., 2006).

**FIGURE 11:**
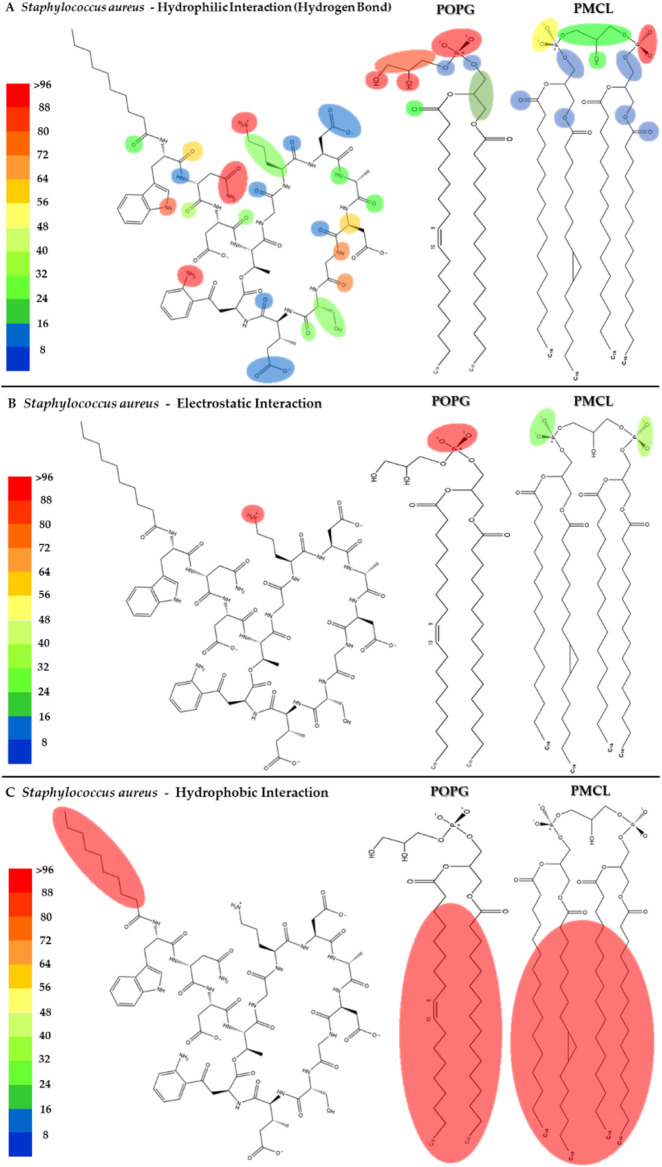
Heatmap of daptomycin interactions with POPG and PMCL1 for the *S. aureus* membrane model. The interaction frequency scale ranges from blue (lowest number of interactions) to red (highest number of interactions) **(A)**: Hydrophilic interactions–*S. aureus*; **(B)** Electrostatic interactions–*S. aureus*; **(C)** Hydrophobic interactions–*S. aureus*.

To visualize the specific contact points for these interactions, Figure 11 shows a heatmap indicating the areas of contact between daptomycin and POPG and PMCL1 in the *S. aureus* membrane model, indicating the hydrogen, electrostatic and hydrophobic intermolecular interactions. Hydrogen bonds were the most common interactions between the functional amino and carbonyl groups in the peptide bonds and the functional side groups of quinurenine and ornithine (Figure 11A). The latter predominantly mediates both hydrogen bonds and electrostatic interaction with POPG phosphates (Figure 11B). Thus, both ORN and POPG are essential for the selectivity of daptomycin toward *S. aureus*, as the formation of pores in the membrane by daptomycin requires the presence of POPG (Baltz, 2009). Thus, the carboxylate groups of other amino acid residues might interact with Ca^2+^ to facilitate daptomycin’s binding to the phospholipids with an adequate adjustment at the binding site of the polar head. This might favorably orient the acyl tail of daptomycin to the hydrophobic nucleus of the membrane (Supplementary Figure S3), facilitating its insertion revealed by the hydrophobic interactions between the decanoic fatty acid of daptomycin and the acyl chains of both phospholipids that make up the membranes of *S. aureus* (Figure 11C). This prediction of daptomycin insertion into *S. aureus* membranes may be closely related to its strong inhibitory activity against this bacterium, as MIC values as low as 0.1 μg/mL have been reported by Louie et al. (Louie et al., 2001), which is associated with its ability to disrupt the integrity of the cytoplasmic membrane (Hobbs et al., 2008). This detailed mapping confirms that multiple residues on daptomycin engage with the *S. aureus* lipids, culminating in the critical insertion of its fatty acid tail.

**FIGURE 12:**
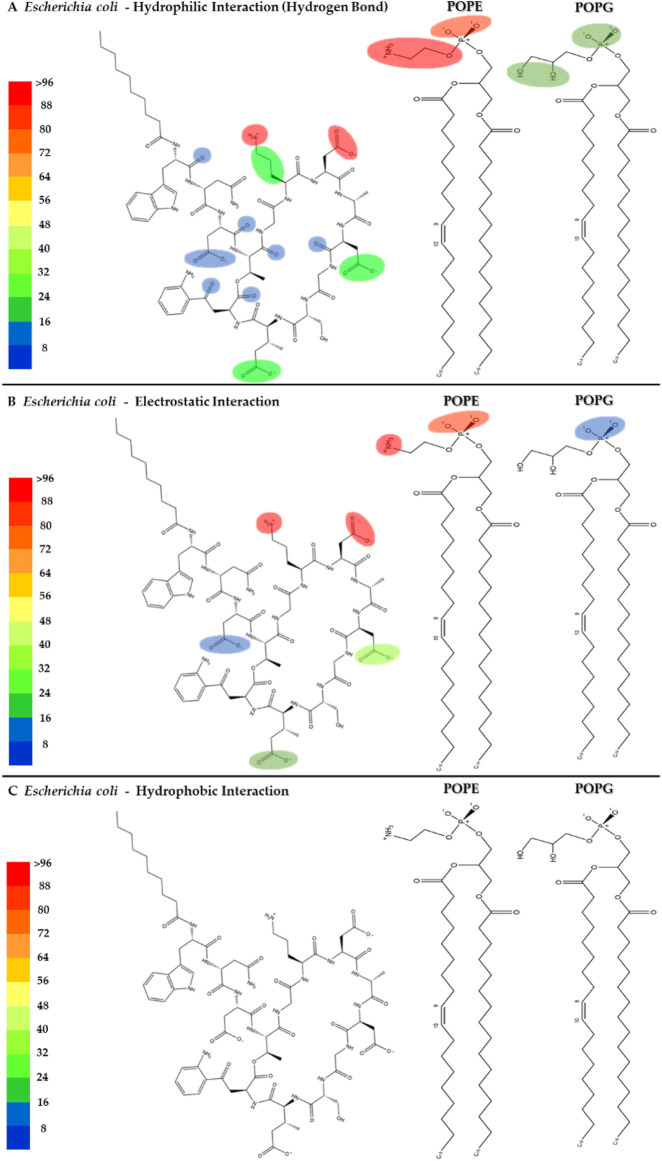
Heatmap of daptomycin’s interactions with POPE and POPG from the *E. coli* membrane model. The interaction frequency scale ranges from blue (lowest number of interactions) to red (highest number of interactions) **(A)**: Hydrophilic interactions–*E. coli*; **(B)** Electrostatic interactions–*E. coli*; **(C)** Hydrophobic interactions–*E. coli*.

The interactions between daptomycin and the *E. coli* membrane model were nearly null in relation to the number of functional groups and the magnitude of the interaction (Figures 12A–C). In contrast to the *S. aureus* model, the electrostatic interactions between the carboxyl groups of aspartic acid (ASP) and the phospholipids in the *E. coli* model are stronger. This increase in interaction strength may be attributed to the displacement of Ca^2+^ ions by the amino groups of POPE. Consequently, this ion displacement affects the conformation of daptomycin, a molecule whose antimicrobial activity is mediated by the Daptomycin-Ca^2+^ complex (Randall et al., 2013). The lack of interaction between daptomycin and the hydrophobic core of the *E. coli* membrane is associated with its weak antimicrobial activity (MIC >256 μg/mL), owing to the permeability barrier imposed by the outer membrane (Randall et al., 2013). These results clearly demonstrate the minimal interaction between daptomycin and the *E. coli* model lipids (Supplementary Figure S4), explaining its lack of activity from a cytoplasmic membrane interaction standpoint.

**FIGURE 13:**
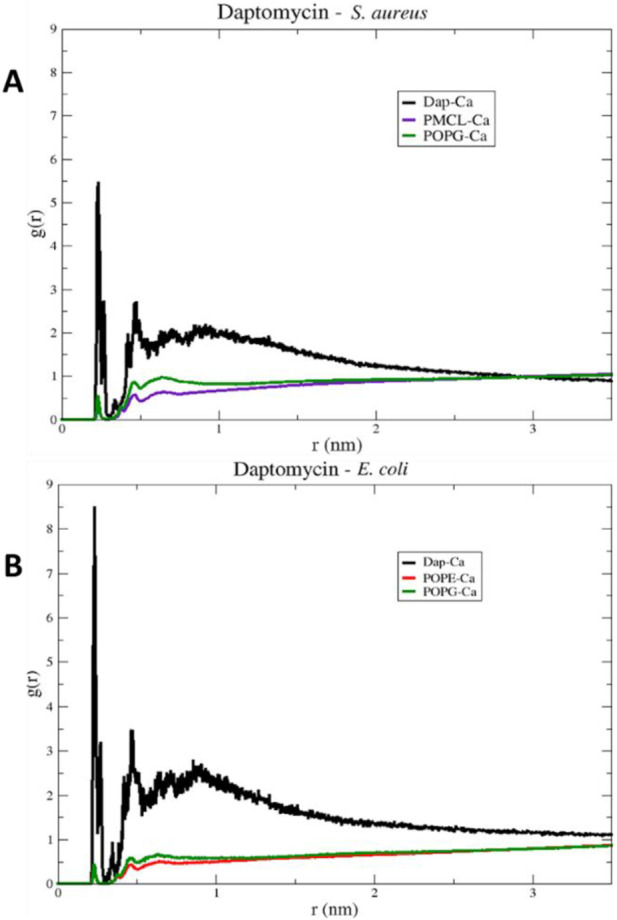
RDF (Radial Distribution Function) of daptomycin in models **(A)** *S. aureus* and **(B)** *E. coli*, for a 100 ns simulation.

To investigate the role of ions in mediating these interactions, in Figure 13, the radial distribution functions for the membrane models of *S. aureus* and *E. coli* are observed. The radial distribution of calcium ions (mobile molecules) around daptomycin and the phospholipids present in each membrane, serving as reference molecules (static molecules), is visualized. Initially, the peaks of greater height in both graphs indicate a higher preference of calcium ions to position themselves around daptomycin for both membrane models, compared to the membrane phospholipids. However, the specificity of calcium ions for daptomycin is more pronounced in the *E. coli* membrane model. This behavior may be attributed to the decrease in the net negative charge of the membrane due to positively charged functional groups, such as the ethanolamine group present in the POPE phospholipid. Thus, this may lead to a reduction of the concentration of calcium ions at the membrane surface affecting the ionic balance suitable for the proper interaction between this lipopeptide and the *E. coli* membrane starting from the fact that Ca^2+^ can act as a bridge between the anionic daptomycin and the headgroups of membrane phospholipids (Straus and Hancock, 2006). When contrasting these radial distribution results of calcium ions around daptomycin with the RMSD results (Figure 8), the effect of calcium ions on the structural stabilization of daptomycin as it approaches the membrane is evident. Therefore, this could limit its permeabilizing action on the bacterial membrane, encountering a potential electrostatic barrier that may repel positively charged ions like calcium. This suggests that the lipid composition of the *E. coli* membrane is less conducive to attracting the Ca^2+^ ions necessary for daptomycin’s function, further contributing to its inactivity against this bacterium.

**FIGURE 14:**
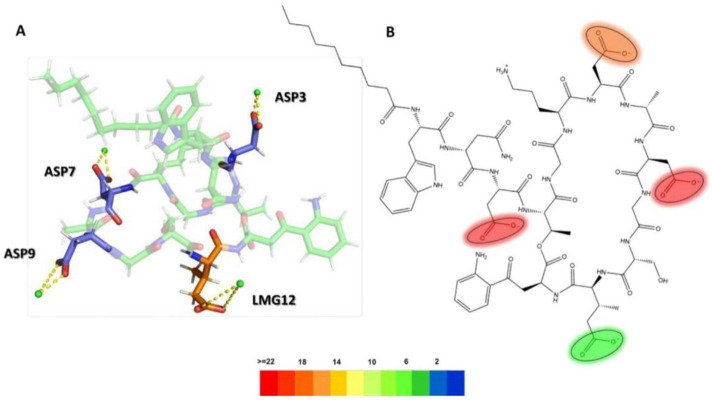
**(A)** 3D representation of Ca^2+^ ion interactions with daptomycin, **(B)** Electrostatic interactions heatmap with Ca^2+^ ions (ASP3: L-aspartic acid 3, ASP7: L-aspartic acid 7, ASP9: L-aspartic acid 9, LMG12: 3-methyl-L-glutamic acid 12).

In order to understand the role of the Ca^2+^ on the interaction between daptomycin and the *S. aureus* membrane, we created a 3D representation of the interactions of Ca^2+^ ions with the deprotonated carboxyl groups of L-aspartic acid residues (ASP3, ASP7, ASP9) and 3-methyl-L-glutamic acid residues (LMG12), along with the interaction frequency for each residue in a 100 ns simulation time (Figure 14). The ASP3, ASP7, and ASP9 residues were primarily involved in stabilizing the daptomycin structure, which contributed to stabilizing its interaction with the bacterial membrane phospholipids, especially in *S. aureus*. In a previous study using mutant analogues of daptomycin, it was concluded that ASP7 and ASP9 are essential for antimicrobial activity (Grünewald et al., 2004). Ho et al. (2008), reported that Ca^2+^ is involved in the aggregation of daptomycin monomers until reaching a micellar form in solution. Thus, they propose that residues Trp1, MeGlu12, and Kyn13, which play a role in the interactions with membrane models described above, are at the intermolecular interface and that the calcium ion serves to neutralize the negative charge between the daptomycin molecules in the micelle, which act as a vehicle to deliver daptomycin to the bacterial cell membranes in high local concentrations and in a functional conformation (Zhang et al., 2016). Thereby, Ca^2+^ serve to mask the negatively charged residues in anionic lipopeptides such as daptomycin, thereby enabling the antibiotic to interact and perturb bacterial membranes in a detergent-like manner. On the other hand, in the *E. coli* membrane model, a positive charge gradient was present due to POPE molecules, which might facilitate displacement of the Ca^2+^ ions in solution, hindering stabilization of the structure responsible for membrane disruption. This analysis highlights the essential role of Ca2+ in bridging daptomycin to the anionic surface of the *S. aureus* membrane, an interaction that is likely hindered at the more zwitterionic surface of the *E. coli* inner membrane.

**FIGURE 15:**
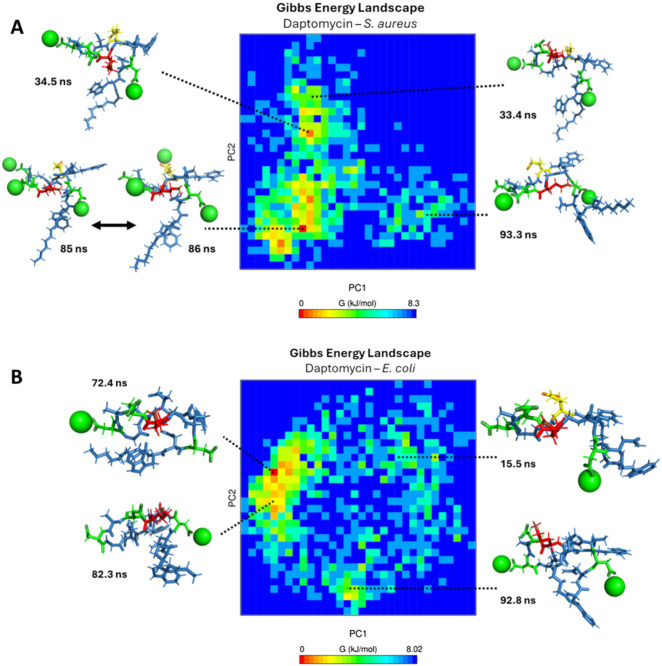
Gibbs free energy diagram calculated by principal component analysis (PCA) **(A)** *S. aureus* membrane model system. **(B)** *E. coli* membrane model system. The green spheres represent calcium ions, the green tubes represent aspartic acid residues, the red tubes represent ornithine residues, and the yellow tubes represent L-methylglutamic acid.

Finally, to visualize the conformational dynamics leading to the most stable states, in Figure 15A, a Gibbs free energy profile diagram is shown by principal component analysis (PCA) to elucidate the structural conformational changes of daptomycin in conjunction with calcium ions on the *S. aureus* membrane model. For the conformations shown at 33.4 ns, 34.5 ns, 85 ns, and 86 ns, the linear chain of daptomycin adopts a perpendicular orientation relative to the cyclic fragment of daptomycin. Moving from left to right along the X-axis of the energy diagram, the orientation of the linear chain changes from a parallel orientation to a perpendicular one with respect to the cyclic structure of daptomycin, as observed in the sampled conformations. In contrast, the variation along the Y-axis reflects local conformational changes in the cyclic structure, primarily concerning the residue ORN6. At the beginning of the PC2 axis, ORN6 is positioned towards ASP3, and as it progresses upward along component PC2, the ORN6 residue moves in the opposite direction to the ASP3 residue. It is evident that the most significant conformational changes occur along the first two principal components (PC1 and PC2), which generate greater variation and effect on the energetic stability change of daptomycin in its interaction with the membrane model. For the most stable conformation, observed at 85 and 86 ns, the residues ASP3 and ASP7 require the inclusion of calcium ions, along with ASP9 and LMG12, which may further stabilize the transition state to the structural conformation with the highest affinity for the membrane model. Secondly, the next most stable structural conformation is observed at 34.5 ns, where the inclusion of calcium ions on ASP3 and ASP7 effectively stabilizes the carboxylate groups which acting as a barrier to electrostatic repulsion during their approach to the negatively charged membrane model.

In Figure 15B, a Gibbs free energy profile diagram is presented using principal component analysis to determine the conformational changes of daptomycin in conjunction with calcium ions on the *E. coli* membrane model. For the four conformations shown, the linear fragment of daptomycin is oriented parallel to the cyclic fragment of daptomycin. Moving from left to right along the X-axis of the energy diagram, the shape of the linear chain transitions from an extended form to a self-folded form, as observed in the sampled conformations. Conversely, the variation along the Y-axis corresponds to local conformational changes in the cyclic structure, including rotations in bond angles and dihedrals. Among the four conformations shown at different simulation times, the proximity of ASP7 to ORN6 consistently appears, as the calcium ions are predominantly repelled by this membrane model in comparison to the *S. aureus* model. This is consistent with the absence of phosphatidylethanolamine, which carries an additional positive charge compared to other membrane phospholipids. With the decreased solvation effect of calcium ions on the membrane surface, interactions between ORN6 and ASP7 become even more prominent due to the inductive effect present on both functional groups (McGee and McLuckey, 2013). The energy landscapes reveal that daptomycin only achieves a low-energy, stable, membrane-inserted state in the presence of the *S. aureus* model lipids, while it remains in higher-energy, non-inserted states when interacting with the *E. coli* model.

In conclusion, for daptomycin, our simulations show that its selectivity towards the *S. aureus* model membrane is mediated by Ca^2+^ ions, which bridge the antibiotic’s anionic residues (ASP, LMG) to the anionic phospholipids (POPG, PMCL1). This stable, ion-mediated binding facilitates the insertion of daptomycin’s lipid tail into the membrane core. Conversely, the zwitterionic nature of the *E. coli* model membrane, with its high POPE content, appears to be less favorable for Ca2+ coordination and subsequent peptide interaction, preventing tail insertion and thus explaining daptomycin’s selectivity for Gram-positive bacteria at the cytoplasmic membrane level.

## 4 Conclusion

In this study, MD simulations were conducted for a duration of 100 ns to obtain detailed information on the interactions between the colistin/daptomycin lipopeptides and the bacterial membrane models of *E. coli* and *S. aureus*. We showed that the type and magnitude of interaction depend on the lipopeptide and phospholipid. In the case of *E. coli*, the DAB and THR of colistin are mainly engaged in non-covalent interactions with POPE, promoting adequate stability for the insertion of the colistin’s acyl tail into the hydrophobic bilayer of the membrane model. However, despite the presence of hydrogen bonds and electrostatic interactions with the membrane surface, the acyl tail of daptomycin could not be inserted into the lipid bilayer, probably due to an inadequate adjustment in relation to the POPE polar head. Further, with regard to *S. aureus*, the tryptophan (TRP1), asparagine (ASN2), ornithine (ORN6), serine (SER11), and kynurenine (KYN13) residues of daptomycin conjugated with Ca^2+^, which stabilized the peptide at the polar head, particularly for POPG, promoting the insertion of the hydrophobic tail into the apolar region of the membrane. Meanwhile, colistin showed weak interactions with both phospholipids, probably because the calcium ions suspended in the solution might repel the positive charges of the side chains of the colistin residues. Based on our simulations, we observed that specific peptide-phospholipid interactions leading to membrane insertion may contribute to the observed selectivity, but acknowledge that this is a simplified model. Additional factors, such as membrane potential and, critically, the presence of lipopolysaccharides or teichoic acids, are likely involved. Therefore, further studies on the influence of these factors on bacterial selectivity, particularly the primary interaction of colistin with LPS, should be considered in future work.

## Data Availability

The datasets presented in this study can be found in online repositories. The names of the repository/repositories and accession number(s) can be found below: http://www.wwpdb.org/, 5L3G; http://www.wwpdb.org/, 1XT7.
