# Supplementary material for: Understanding the selectivity in silico of colistin and daptomycin toward gram-negative and gram-positive bacteria, respectively, from the interaction with membrane phospholipids
Source: Front Bioinform. 2025 Jul 17;5:1569480. doi: 10.3389/fbinf.2025.1569480 (PMC12310579; doi:10.3389/fbinf.2025.1569480)
Supplement: Supplementary file 1 [file DataSheet1.pdf]

Supporting Materials for:

Understanding the selectivity *in silico* of colistin and daptomycin toward Gram-negative and Gram-positive bacteria, respectively, from the interaction with membrane phospholipids

Yesid Aristizabal [1], Yamil Liscano [2]* and José Oñate-Garzón [1]*

[1] Grupo de investigación en química y biotecnología (QUIBIO), Facultad de Ciencias Básicas, Universidad Santiago de Cali, Cali 760035, Colombia. jose.onate00@usc.edu.co (J.O-G) yesid.aristizabal00@usc.edu.co (Y.A)
[2] Grupo de Investigación en salud Integral, Facultad de Salud, Universidad Santiago de Cali, Cali 760035, Colombia. yamil.liscano00@usc.edu.co (Y.L.)
***** Correspondence: yamil.liscano00@usc.edu.co; jose.onate00@usc.edu.co

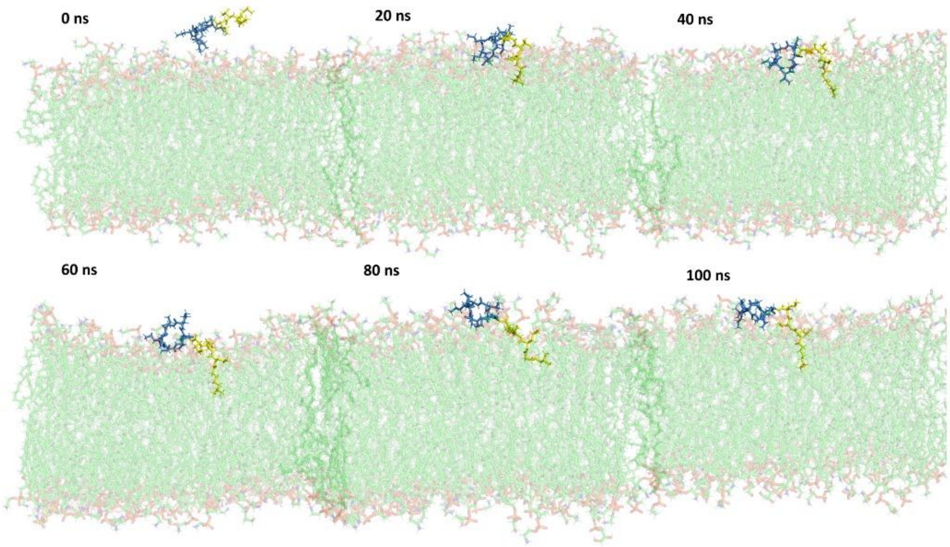

Figura S1. Simulation visualization for colistin in *E. coli* bacterial model during 100 ns

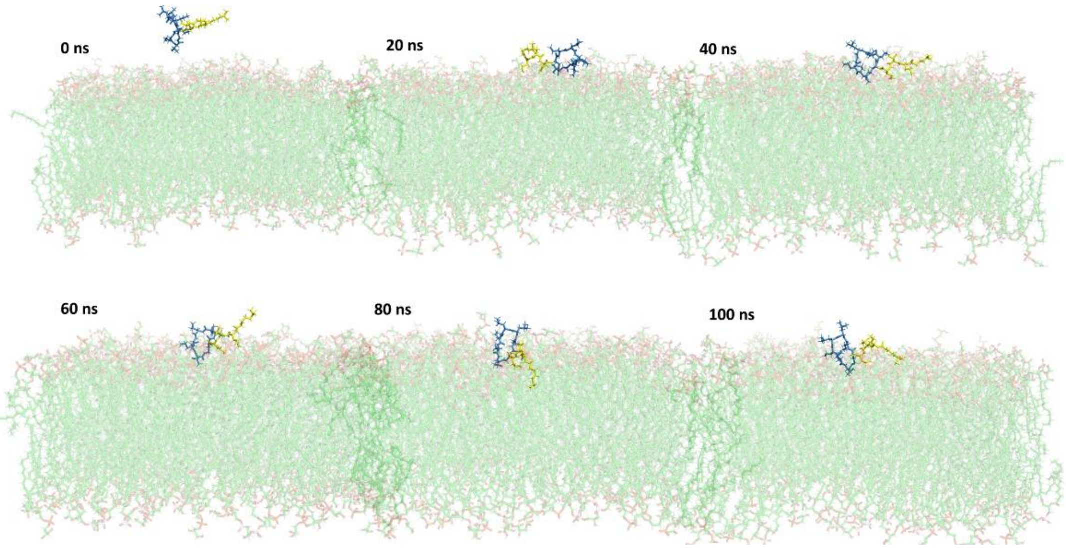

Figura S2. Simulation visualization for colistin in *S. aureus* bacterial model during 100 ns

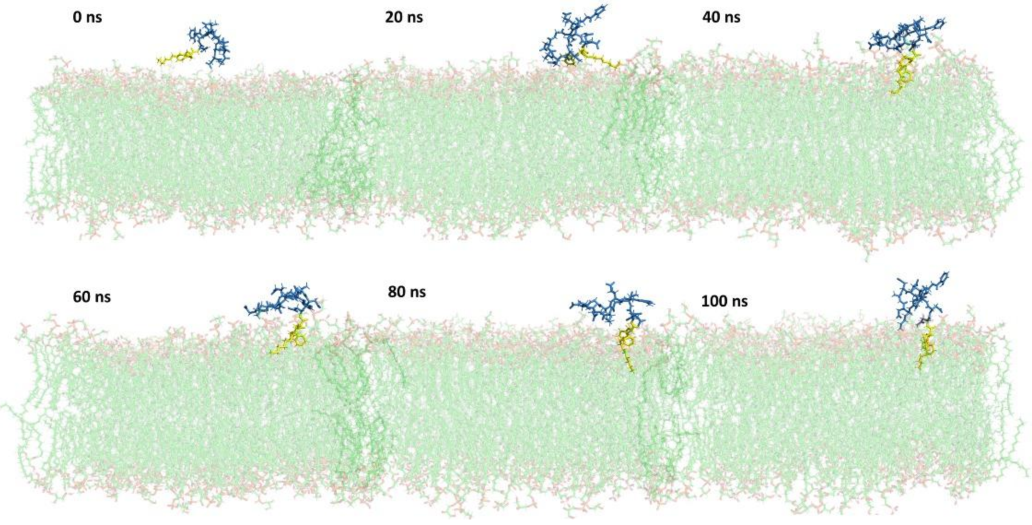

Figura S3. Simulation visualization for daptomycin in *S. auerus* bacterial model during 100 ns

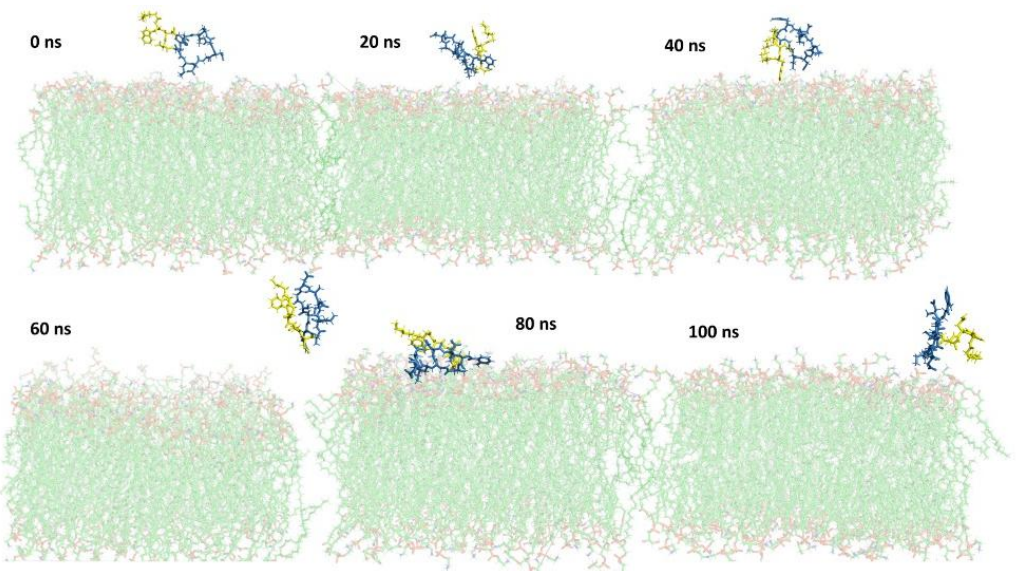

Figura S4. Simulation visualization for daptomycin in *E. coli* bacterial model during 100 ns
